# SGLT2 Inhibitors and External Genital Infection in Male Patients With Type 2 Diabetes

**DOI:** 10.1001/jamanetworkopen.2025.34485

**Published:** 2025-09-29

**Authors:** Tsung-Han Cheng, Kun-Che Lin, Albert Tzu-Ming Chuang, Michael Chun-Yuan Cheng, Shih-Chieh Shao, Swu-Jane Lin, Tsung-Yen Lin, Yuh-Shyan Tsai, Edward Chia-Cheng Lai, Che-Yuan Hu, Kuan-Yu Wu

**Affiliations:** 1 Division of Urology, Department of Surgery, Kaohsiung Show Chwan Memorial Hospital, Kaohsiung, Taiwan; 2 Department of Urology, National Cheng Kung University Hospital, College of Medicine, National Cheng Kung University, Tainan, Taiwan; 3 School of Pharmacy, Institute of Clinical Pharmacy and Pharmaceutical Sciences, College of Medicine, National Cheng Kung University, Tainan, Taiwan; 4 Population Health Data Center, National Cheng Kung University, Tainan, Taiwan; 5 Department of Pharmacy, Keelung Chang Gung Memorial Hospital, Keelung, Taiwan; 6 Department of Pharmacy Systems, Outcomes & Policy, College of Pharmacy, University of Illinois at Chicago, Chicago; 7 National Cheng Kung University Hospital Douliu Branch, Yunlin, Taiwan; 8 Institute of Clinical Medicine, College of Medicine, National Cheng Kung University, Tainan, Taiwan

## Abstract

**IMPORTANCE:**

New medications for type 2 diabetes (T2D) control, such as sodium-glucose cotransporter-2 inhibitors (SGLT2Is) and glucagon-like peptide-1 receptor agonists (GLP-1RAs), have emerged. However, the US Food and Drug Administration has warned about potential risks of Fournier gangrene, a severe genital infection associated with SGLT2I use.

**OBJECTIVE:**

To determine comparative risks of male external genital infections (MEGIs) in adult male patients with T2D treated with SGLT2Is.

**DESIGN, SETTING, AND PARTICIPANTS:**

A population-based cohort study analyzing Taiwan’s National Health Insurance Research Database was conducted to evaluate the MEGI risk in adult male patients with T2D newly treated with SGLT2Is between January 1, 2009, and December 31, 2020. Patients newly treated with GLP-1RAs were chosen during the same period for the active-comparator group, and propensity scores with inverse probability of treatment weighting were applied to balance the baseline characteristics between the 2 treatment groups. Data analyses were conducted between February 2023 and April 2025.

**EXPOSURES:**

Treatment with SGLT2Is or GLP-1RAs.

**MAIN OUTCOMES AND MEASURES:**

The primary outcome was the incidence of MEGI, identified using *International Classification of Diseases, Tenth Revision, Clinical Modification* diagnosis codes. To examine the result consistency, subgroup analyses based on age (older or younger than 60 years), diabetes severity (insulin use or not), and kidney function were conducted.

**RESULTS:**

The study included 239 757 patients; 224 360 initiated SGLT2Is (mean [SD] age, 58.4 [12.3] years) and 15 397 initiated GLP-1RAs (mean [SD] age, 58.1 [13.6] years). Compared with the GLP-1RA group, the SGLT2I group had a significantly increased risk of MEGI (hazard ratio [HR], 1.65; 95% CI, 1.59-1.71). The subgroup analyses indicated an increased risk of MEGI in the SGLT2I group among patients younger than 60 years (HR, 2.04; 95% CI, 1.58-2.65), those with an estimated glomerular filtration rate of 60 mL/min/1.73 m^2^ (HR, 1.69; 95% CI, 1.34-2.15), and those with hemoglobin A_1c_ below 7% (HR, 3.22; 95% CI, 1.71-6.03).

**CONCLUSIONS AND RELEVANCE:**

These findings suggest that SGLT2Is are associated with a higher risk of MEGI in adult male patients with T2D, compared with GLP-1RAs. Clinicians should be cautious when prescribing SGLT2Is in male patients with T2D.

## Key Points

**Question:**

Among adults with type 2 diabetes (T2D), is the use of sodium-glucose cotransporter 2 inhibitors (SGLT2Is) associated with higher risk of male external genital infection (MEGI) than glucagon-like peptide-1 receptor agonists (GLP-1RAs)?

**Findings:**

In this cohort study with 239 757 participants, initiation of SGLT2Is was associated with a higher risk of MEGI compared with initiation of GLP-1RAs in patients with T2D. The increased risk of MEGI with the use of SGLT2Is was observed across different subgroup analyses.

**Meaning:**

These findings suggest that patients with T2D newly receiving SGLT2Is are at a higher risk of MEGI compared with those receiving GLP-1RAs, and clinicians should be cautious when prescribing SGLT2Is to male patients with T2D.

## Teaser

This cohort study investigates the comparative risks of male external genital infections in adult male patients with type 2 diabetes treated with sodium-glucose cotransporter 2 (SGLT2) inhibitors vs glucagon-like peptide-1 receptor agonists (GLP-1RAs).

## Introduction

Diabetes is a chronic metabolic disease characterized by hyperglycemia due to insulin insufficiency. The International Diabetes Federation projects a global diabetes prevalence of 578 million by 2030 and 700 million by 2045.^1^ The primary aim of treating type 2 diabetes (T2D) is to prevent complications, including neuropathy, nephropathy, and retinopathy. Additionally, hyperglycemia can compromise the immune system, increasing the risk of infections, hospitalizations, and death.^2^ A large retrospective cohort study^3^ found that 6% of infection-related hospitalizations and 12% of infection-related deaths were attributable to diabetes, highlighting the importance of infection prevention and control to reduce mortality in patients with T2D.

New medications for diabetes control have been developed, such as sodium-glucose cotransporter-2 inhibitors (SGLT2Is) and glucagon-like peptide-1 receptor agonists (GLP-1RAs). SGLT2Is reduce hyperglycemia by decreasing glucose reabsorption and promoting urinary glucose excretion.^4^ Studies have shown benefits in glycemic control and various comorbidities such as heart failure, dry eye disease, chronic kidney disease, and atherosclerotic cardiovascular disease.^5,6,7,8^ By contrast, GLP-1RAs are synthetic analogs of native GLP-1 and work by enhancing pancreatic β-cell secretion, delaying gastric emptying, and increasing insulin uptake in the peripheral tissues.^9^ Some GLP-1RAs have been linked to reduced cardiovascular events, improved cardiovascular risk factors, and potential nephroprotective effects.^10,11,12^

However, the US Food and Drug Administration (FDA) has warned about the potential risk of Fournier gangrene (FG), a severe genital infection with high mortality, associated with SGLT2Is use.^13,14,15^ Between March 2013 and May 2018, 7 cases of FG were reported among men using SGLT2Is in the US, compared with only 6 cases with other glucose-lowering drugs over more than 30 years.^16,17^ Numerous studies^18,19,20,21,22^ have indicated an association between SGLT2Is and FG, although the exact pathophysiological mechanisms remain unclear, possibly involving glucosuria, immunosuppression, and enhanced bacterial adhesion to the urothelium. A large US cohort study^23^ found an increased, but not statistically significant, hazard ratio (HR) for FG risk among patients taking SGLT2Is compared with dipeptidyl peptidase 4 inhibitors (HR, 1.73; 95% CI, 0.87-3.42), which further increased when GLP-1RAs were used as the comparator (HR, 2.52; 95% CI, 0.91-6.99). Similarly, a study by Mascolo et al^24^ reported a higher FG incidence among patients with diabetes than those with heart failure taking SGLT2Is, suggesting that diabetes itself may be a key risk factor.

Moreover, concerns about genital-urinary infections including urinary tract infections (UTIs), asymptomatic bacteriuria, and nonsexually transmitted genital infections have been raised due to the potential for increased glucosuria facilitated by SGLT2Is.^25,26,27^ Studies have suggested a possible link between SGLT2Is and UTIs, including urethritis, cystitis, pyelonephritis, and, in severe cases, urosepsis.^16^ However, research addressing a specific spectrum of infections involving the external genitalia remains scarce. In male individuals, these infections require heightened attention due to hygiene challenges and limited blood and oxygen supply in this area, and often necessitate surgical intervention for definitive treatment. This study aimed to evaluate the risk of male external genital infections (MEGIs) associated with SGLT2Is, utilizing a nationwide claims database.

## Methods

### Data Sources

This cohort study used Taiwan’s National Health Insurance Research Database (NHIRD), a nationwide claims-based database covering nearly all citizens,^28,29^ and the Chang Gung Research Database (CGRD), which includes laboratory data such as glycemic and kidney function indicators, to assess T2D severity.^8,30,31^ The CGRD is a valuable resource that has previously provided clinical evidence for pharmacoepidemiologic studies.^31,32,33,34^ Most importantly, it includes laboratory information, such as glycemic and kidney function data, enabling a more accurate assessment of T2D severity and yielding results for comparative drug effectiveness research.^8,30,35^ We, therefore, used CGRD to validate our main findings by incorporating laboratory data and disease severity to repeat our primary analysis.^36,37^

The study protocol was approved by the institutional review boards of Chang Gung Medical Foundation and National Cheng Kung University Hospital, with the informed consent requirement waived. All data were anonymized in compliance with approved guidelines and the Declaration of Helsinki. We adhered to the Strengthening the Reporting of Observational Studies in Epidemiology (STROBE) reporting guideline in presenting our findings.

### Study Design and Participants

We defined MEGIs to include balanoposthitis, scrotal infections, epididymo-orchitis, and FG. We examined whether the use of SGLT2Is or GLP-1RAs in patients with T2D is associated with an increased risk of developing MEGI. To avoid self-inflicted biases, we implemented a target trial emulation framework to assess the degree of potential causality between use of SGLT2Is or GLP-1RAs and MEGI risk in male patients with T2D (eTable 1 in Supplement 1).

Eligible participants were adult male patients newly prescribed SGLT2Is (empagliflozin, dapagliflozin, or canagliflozin) or GLP-1RAs (liraglutide or dulaglutide) between 2009 and 2020. GLP-1RAs were selected as the comparator given their similar cardiovascular and kidney benefits, but lack of glucosuria-associated effects.^38^ We defined the first date of use of the SGLT2Is or GLP-1RAs as the index date and considered the year preceding the index date as the baseline period. We excluded nonmale patients and those who had incomplete sex information, were younger than 20 years, or had initiated treatment before 2017. Additionally, we excluded patients with abnormal immune status, such as those with a history of organ transplantation or congenital immunodeficiency. We also excluded patients who received a diagnosis of MEGI within 1 year before the index date (eFigures 1, 2, and 3 in Supplement 1). All statistical analyses were conducted between February 2023 and April 2025.

### Study Outcome

The primary outcome was the incidence of MEGI among users of SGLT2Is or GLP-1RAs. To ensure the validity of the outcome, we identified MEGI using *International Classification of Diseases, Ninth Revision, Clinical Modification (ICD-9-CM) *and *International Classification of Diseases, Tenth Revision, Clinical Modification *(*ICD-10-CM*) clinical diagnosis codes for FG (*ICD-9-CM*, 608.83 and 608.4; *ICD-10-CM*, N493), scrotal abscess (*ICD-9-CM*, 608.4; *ICD-10-CM*, N492), epididymitis and/or orchitis (*ICD-9-CM*, 604.90, 604.91, and 604.99; *ICD-10-CM*, N451, N452, and N453), abscess of the testis and/or epididymis (*ICD-9-CM*, 604.0; *ICD-10-CM*, N454), and balanoposthitis (*ICD-9-CM*, 607.1; *ICD-10-CM*, N476, N477, N481, and B3742). In Taiwan’s National Health Insurance system, the same *ICD-10-CM* diagnosis codes are applied uniformly across both inpatient and outpatient settings, ensuring consistency in diagnostic classification. Although formal validation studies specific to MEGI-related codes are limited, the overall reliability of NHIRD-based outcomes has been supported by validation efforts demonstrating high positive predictive values (typically ranging from 80% to 99%) for many common diagnoses, including diabetes.^39,40^ We followed patients from the index date until the first occurrence of MEGI, death, or the end of the database period (December 31, 2022). Data analysis was conducted from February 1, 2023, to March 31, 2025.

### Covariates

We used a 1-year lookback period to assess covariates, including demographics, comorbidities, and comedications. We selected baseline comorbidities related to MEGI, such as obesity, peripheral vascular disease, chronic kidney disease, and liver failure, and considered major adverse cardiac events, including myocardial infarction, stroke, and unstable angina, because major adverse cardiac events may be related to the likelihood of patients being assigned to either group.^41^ We also included other chronic diseases related to inflammation status, such as benign prostatic hypertrophy, asthma, chronic obstructive pulmonary disease, arthritis, gout, rheumatoid disease, and thyroid disorders. We used the Charlson Comorbidity Index composite scores to assess the overall disease burden.^42^

### Statistical Analysis

We summarized age as mean (SD), laboratory values as median (IQR), and categorical variables as counts and percentages. Group balance was assessed using absolute standardized mean differences.^48^ We employed Cox proportional hazards models to estimate HRs and 95% CIs for MEGI risks, using GLP-1RAs as the reference group. All analyses were performed using SAS statistical software version 9.4 (SAS Institute). Statistical significance was set at *P *< 0.05 and were 2-sided.

#### Propensity Score Weighting

To reduce baseline differences and simulate randomization, we used inverse probability of treatment weighting (IPTW) based on propensity scores. This method creates a weighted pseudopopulation with balanced covariates between treatment groups, mimicking a target trial. Unlike propensity score matching, which limits analysis to matched pairs and estimates the average treatment effect for the treated, IPTW retains the full cohort and estimates the average treatment effect, thus enhancing generalizability and statistical power. We calculated weights using logistic regression based on demographic and clinical covariates. This method allows for better adjustment of confounding and enables population-level comparisons without reducing the sample size through matching.^43,44^ To improve covariate balance and reduce the impact of extreme weights, we applied IPTW with trimming at the 2.5th and 97.5th percentiles of the propensity score distribution.

#### Subgroup and Sensitivity Analyses

To clarify MEGI risk differences between SGLT2Is and GLP-1RAs, we performed IPTW-adjusted subgroup analyses by age (<60 vs ≥60 years) and baseline insulin use. We performed multiple sensitivity analyses to assess the robustness of our findings. Using 1:1 propensity score matching with greedy nearest-neighbor matching (without replacement), we progressively relaxed the matching precision from 8-digit to 1-digit to optimize match rates. This aimed to emulate a randomized trial by balancing covariates between treatment groups.^45,46^ We conducted a during-treatment analysis to evaluate the association between treatment discontinuation or medication switching and the primary outcome. Discontinuation was defined as a gap of more than 90 days from the last prescription fill date, regardless of the days of supply. In the during-treatment analysis, patients were followed from the index date until discontinuation, switching to another group, their last clinical visit, death, or the end of the database, whichever occurred first. To assess temporal effects, we recalculated MEGI incidence and hazard ratios (HRs) at 1-year, 2-year, and 3-year intervals, using the same initial cohort for consistency. Finally, we performed E-value analysis to evaluate the potential impact of unmeasured confounding.

#### Analysis Using CGRD

To validate the robustness of the primary outcome and incorporate variables unavailable in the NHIRD, we repeated the analysis using the CGRD, integrating hemoglobin A_1c_ (HbA_1c_) and estimated glomerular filtration rate (eGFR) into the propensity score model. As noted by Tsai et al,^47^ Taiwan benefits from a dual infrastructure of claims data and hospital-based electronic health record systems (ie, CGRD). We further conducted subgroup analyses stratified by baseline HbA_1c_ (<7% vs ≥7%; to convert to proportion of total hemoglobin, multiply by 0.01) and eGFR (<60 vs ≥60 mL/min/1.73 m^2^) to assess potential effect modification. Additionally, to explore possible biological mechanisms, changes in HbA_1c_ and eGFR over time were evaluated using complete case analysis.

## Results

### Baseline Characteristics

The study included 239 757 male patients with T2D, 224 360 of whom were newly prescribed SGLT2Is (empagliflozin, dapagliflozin, or canagliflozin) and 15 397 of whom were newly prescribed GLP-1RAs (liraglutide or dulaglutide) from 2009 to 2020. Before IPTW, the mean (SD) age was similar between the SGLT2I and GLP-1RA groups (mean [SD] age, 58.4 [12.3] vs 58.1 [13.6] years). The SGLT2I group had a lower prevalence of obesity (4212 patients [1.9%] vs 584 patients [3.8%]), chronic kidney disease (15 408 patients [6.9%] vs 2987 patients [19.4%]), and insulin use (52 769 patients [23.5%] vs 10 036 patients [65.2%]), but had a higher prevalence of metformin (203 737 patients [90.8%] vs 11 886 patients [77.2%]) and dipeptidyl peptidase-4 inhibitor (DPP4I) (139 586 patients [62.2%] vs 11 439 patients [74.3%]) prescriptions compared with the GLP-1RAs group (absolute standardized mean difference >0.1). After IPTW, the number of patients in the SGLT2I group was 239 747, and that in the GLP-1RA group was 235 706. All baseline characteristics, including age, comorbidities, major adverse cardiac events, and diabetes comedications were well balanced (Table 1).

**Table 1.** Baseline Characteristics Before and After IPTW, Taiwan National Health Insurance Research Database

Characteristic	Patients, No. (%)					
	Before IPTW			After IPTW		
	SGLT2Is (n = 224 360)	GLP-1RAs (n = 15 397)	ASMD^a^	SGLT2Is (n = 239 747)	GLP-1RAs (n = 235 706)	ASMD^a^
Age, mean (SD), y	58.4 (12.3)	58.1 (13.6)	0.027	58.4 (12.8)	57.1 (51.4)	0.03
Age group, y						
20-39	16 818 (7.5)	1612 (10.5)	−0.104	18 495 (7.7)	23 212 (9.8)	0.08
40-59	102 194 (45.5)	6644 (43.2)	0.048	108 820 (45.4)	109 873 (46.6)	0.03
60-79	97 319 (43.4)	6358 (41.3)	0.042	103 615 (43.2)	93 695 (39.8)	0.07
≥80	8029 (3.6)	783 (5.1)	−0.074	8818 (3.7)	8927 (3.8)	<0.01
Comorbidities						
Elevated prostate-specific antigen level	1195 (0.5)	79 (0.5)	0.003	1277 (0.5)	1535 (0.7)	0.02
Benign prostate hyperplasia	30 607 (13.6)	2260 (14.7)	−0.03	32 884 (13.7)	33 229 (14.1)	0.01
Congestive heart failure	11 346 (5.1)	972 (6.3)	−0.054	12 292 (5.1)	12 058 (5.1)	<0.01
Asthma	9258 (4.1)	735 (4.8)	−0.031	9999 (4.2)	10 081 (4.3)	<0.01
Chronic obstructive pulmonary disease	8908 (4)	736 (4.8)	−0.04	9650 (4)	10 610 (4.5)	0.02
Alcohol abuse	348 (0.2)	21 (0.1)	0.005	370 (0.2)	598 (0.3)	0.02
Peripheral vascular disease	2389 (1.1)	222 (1.4)	−0.034	2608 (1.1)	2367 (1)	<0.01
Hypertension	118 938 (53)	8513 (55.3)	−0.046	127 407 (53.1)	124 454 (52.8)	<0.01
Obesity	4212 (1.9)	584 (3.8)	−0.116	4837 (2)	5534 (2.3)	0.02
Chronic kidney disease	15 408 (6.9)	2987 (19.4)	−0.378	18 285 (7.6)	15 631 (6.6)	0.04
Coagulopathy	152 (0.1)	20 (0.1)	−0.02	170 (0.1)	118 (0.1)	<0.01
Liver disease	33 294 (14.8)	2132 (13.8)	0.028	35 447 (14.8)	37 272 (15.8)	0.03
Liver failure	1446 (0.6)	127 (0.8)	−0.021	1569 (0.7)	1614 (0.7)	<0.01
Arthritis	1422 (0.6)	112 (0.7)	−0.011	1538 (0.6)	1769 (0.8)	0.01
Gout	22 583 (10.1)	1834 (11.9)	−0.059	24 425 (10.2)	24 216 (10.3)	<0.01
Rheumatoid diseases	231 (0.1)	19 (0.1)	−0.006	249 (0.1)	248 (0.1)	0.00
Thyroidism	2864 (1.3)	240 (1.6)	−0.024	3112 (1.3)	3257 (1.4)	<0.01
Major adverse cardiac events						
Myocardial infarction	8209 (3.7)	469 (3)	0.034	8659 (3.6)	8006 (3.4)	0.01
Stroke	16 754 (7.5)	1382 (9)	−0.055	18 126 (7.6)	18 355 (7.8)	<0.01
Unstable angina	2334 (1)	167 (1.1)	−0.004	2497 (1)	2033 (0.9)	0.02
Antidiabetic medications						
Metformin	203 737 (90.8)	11 886 (77.2)	0.378	215 739 (90)	215 747 (91.5)	0.05
Sulfonylurea	140 299 (62.5)	9479 (61.6)	0.020	149 857 (62.5)	154 619 (65.6)	0.06
Dipeptidyl peptidase–4 inhibitor	139 586 (62.2)	11 439 (74.3)	−0.262	151 039 (63)	154 292 (65.5)	0.05
Thiazolidinedione	45 742 (20.4)	3718 (24.1)	−0.090	49 540 (20.7)	54 649 (23.2)	0.06
α-Glucosidase inhibitor	34 616 (15.4)	3246 (21.1)	−0.147	37 916 (15.8)	41 309 (17.5)	0.05
Glinide	391 (0.2)	47 (0.3)	−0.027	438 (0.2)	447 (0.2)	<0.01
Insulin	52 769 (23.5)	10 036 (65.2)	−0.924	62 810 (26.2)	64 245 (27.3)	0.02

Abbreviations: ASMD, absolute standardized mean difference; GLP-1RAs, glucagon-like peptide-1 receptor agonists; IPTW, inverse probability of treatment weighting; SGLT2Is, sodium-glucose cotransporter-2 inhibitors.

^a^ ASMDs greater than 0.1 suggest a meaningful difference between the 2 treatment groups.

### Main Outcomes

Crude analysis showed MEGI incidence rates of 10.90 cases per 1000 person years for the GLP-1RA group and 16.34 cases per 1000 person-years for the SGLT2I group (HR, 1.45; 95% CI, 1.30-1.65). The mean (SD) follow-up duration was 1.69 (x) years for the GLP-1 RA group and 1.97 (x) years for the SGLT2I group, providing context for the subsequent analyses stratified by 1-year, 2-year, and 3-year follow-up. After IPTW, the incidence rates were 9.67 cases per 1000 person-years for the GLP-1RA group and 16.33 cases per 1000 person-years for the SGLT2I group (HR, 1.65; 95% CI, 1.59-1.71) (Table 2). The number needed to harm was 150, indicating that 1 additional case of MEGI may occur for every 150 patients treated with SGLT2Is instead of GLP-1RAs. To further clarify the types of infections included in MEGI, we analyzed the distribution of infection subtypes. In the GLP-1RA group, cases included 3 of FG, 11 of balanoposthitis, and 14 of scrotal abscess or epididymo-orchitis. In contrast, the SGLT2I group reported 22 cases of FG, 204 cases of balanoposthitis, and 137 cases of scrotal abscess or epididymo-orchiti. The Figure presents the cumulative incidence of MEGI in the 2 groups over time.

**Table 2.** Risk of Male External Genital Infection for GLP-1RAs vs SGLT2Is, Taiwan National Health Insurance Research Database

Variable	Patients, No.	Incidence rate, No. of cases/1000 person-years (95% CI)	HR (95% CI)
Crude analysis			
GLP-1RAs	15 397	10.90 (9.75-12.14)	1.00 [Reference]
SGLT2Is	224 360	16.34 (15.94-16.76)	1.45 (1.30-1.65)
Main analysis (inverse probability of treatment weighting)			
GLP-1RAs	235 706	9.67 (9.40-9.94)	1.00 [Reference]
SGLT2Is	239 747	16.33 (15.93-16.73)	1.65 (1.59-1.71)

Abbreviations: GLP-1RAs, glucagon-like peptide-1 receptor agonists; HR, hazard ratio; SGLT2Is, sodium-glucose cotransporter-2 inhibitors

**Figure.**
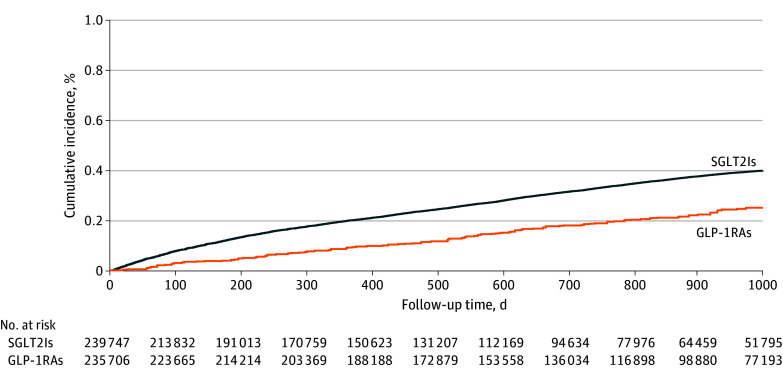
Cumulative Incidence of Male External Genital Infections for SGLT2Is and GLP-1RAs After Inverse Probability of Treatment Weighting Abbreviations: GLP-1RAs, glucagon-like peptide-1 receptor agonists; SGLT2Is, sodium-glucose cotransporter-2 inhibitors.

### Subgroup and Sensitivity Analyses

Subgroup analyses showed higher MEGI incidence in the SGLT2I group vs GLP-1RA group regardless of age or baseline insulin use, with a greater HR in those not using insulin (HR, 1.81; 95% CI, 1.73-1.89) (Table 3). However, the HRs across age groups were identical with nearly overlapping 95% CIs. We observed similar trends in the sensitivity analyses (eTable 2 and eTable 3 in Supplement 1). A higher risk of MEGI was noted in the SGLT2I group, including the during-treatment analysis (HR, 2.07; 95% CI, 1.55-2.76) and across different follow-up periods, with a 1-year HR of 1.22 (95% CI, 0.91-1.62), a 2-year HR of 1.29 (95% CI, 1.02-1.62), and a 3-year HR of 1.34 (95% CI, 1.09-1.64). E-value analysis showed that an unmeasured confounder would need a risk ratio of at least 2.69 with both treatment and outcome to nullify the primary HR of 1.65 (eFigure 6 in Supplement 1). Forest plots from sensitivity analyses are presented in eFigure 4 in Supplement 1, and forest plots from CGRD analyses are shown in eFigure 5 in Supplement 1.

**Table 3.** Subgroup Analyses of Risk of Male External Genital Infection Associated With SGLT2Is vs GLP-1RAs

Variable	Patients, No.	Incidence rate, No. of cases/1000 person-years (95% CI)	HR (95% CI)
Age			
<60 y			
GLP-1RAs	133 084	1.25 (1.21-1.29)	1.00 [Reference]
SGLT2Is	127 314	2.18 (2.12-2.24)	1.69 (1.63-1.77)
≥60 y			
GLP-1RAs	102 622	0.55 (0.52-0.58)	1.00 [Reference]
SGLT2Is	112 433	0.95 (0.91-1.00)	1.69 (1.57-1.82)
Insulin usage at baseline			
No			
GLP-1RAs	171 461	0.90 (0.87-0.93)	1.00 [Reference]
SGLT2Is	176 937	1.68 (1.63-1.72)	1.81 (1.73-1.89)
Yes			
GLP-1RAs	64 245	1.17 (1.11-1.22)	1.00 [Reference]
SGLT2Is	62 810	1.51 (1.43-1.58)	1.28 (1.19-1.37)

Abbreviations: GLP-1RAs, glucagon-like peptide-1 receptor agonists; HR, hazard ratio; SGLT2Is, sodium-glucose cotransporter-2 inhibitors

### Analysis From the CGRD

CGRD data had a mean (SD) follow-up time of 2.79 (x) years. After IPTW, MEGI incidence was higher in the SGLT2I group than in the GLP-1RA group (3.63 cases per 1000 person-years vs 2.32 per 1000 person-years; HR, 1.55; 95% CI, 1.28-1.89) (eTable 3 in Supplement 1). In the subgroup analyses, we observed MEGI to be more pronounced in younger patients (HR, 2.04; 95% CI, 1.58-2.65), in those with better kidney function (HR, 1.69; 95% CI, 1.34-2.15), and in those with HbA_1c_ less than 7% (HR, 3.22; 95% CI, 1.71-6.03) (eTable 4 in Supplement 1). HbA_1c_ and eGFR changes over time were similar between groups (eTable 5 in Supplement 1).

## Discussion

In this population-based cohort study, we found that SGLT2Is were associated with a higher risk of MEGI, compared with GLP-1RAs, among male patients with T2D. The HR for MEGI formation was 1.65 (95% CI, 1.59-1.71). The cumulative incidence of MEGI increased over a 3-year follow-up period with prolonged medication use. Moreover, regardless of age, kidney function, and diabetes control, the risk of MEGI was consistently higher in the SGLT2I group than in the GLP-1RA group. Additionally, the comparable levels of glycemic control and changes in kidney function observed in both the SGLT2I and GLP-1RA groups suggest that the mechanisms contributing to the increased incidence of MEGI with SGLT2I use are independent of these factors. The results from sensitivity analyses remained consistent across various statistical approaches, follow-up periods, and databases. On the basis of these findings, we concluded that SGLT2Is may carry a higher risk of MEGI than GLP-1RAs in patients with T2D. The estimated number needed to harm was 150, which provides a clinically interpretable measure of risk and may aid in shared decision-making. Clinicians should counsel patients with T2D about the potentially increased risks of MEGI associated with the use of SGLT2Is.

SGLT2Is manage glycemic control by inhibiting glucose reabsorption, thereby increasing glucose excretion to urine via the kidneys.^49^ Glucosuria has been proposed as a key mechanism underlying the increasing risk of UTIs. Furthermore, in 2015, the FDA announced that SGLT2Is might elevate the risk of serious UTIs.^16^ However, the association between SGLT2Is and UTIs remains controversial. Some studies^50,51,52^ have demonstrated an association between the use of SGLT2Is and an increased risk of UTIs. Conversely, many studies^5,7,53,54,55,56,57,58,59,60^ have found no significant difference in UTI prevalence when comparing SGLT2Is with placebos or other antiglycemic medications. Therefore, it remains a matter for debate whether patients with T2D who are at risk of complicated UTIs should discontinue SGLT2Is.

In addition to UTIs, SGLT2Is have been linked to an increased risk of genital infections, including mycotic infections^61,62,63^ and the rare but life-threatening genital infection, FG, prompting an FDA warning in 2018. A study by Mei et al^21^ identified 542 FG cases linked to SGLT2Is use in the US FDA Adverse Events Reporting System database but could not estimate incidence due to limited clinical data. Our study aimed to address this gap by focusing on the incidence rate of FG and other external genital infections in male patients with T2D using SGLT2Is. Additionally, we used GLP-1RAs, another new antidiabetic medication class that offers similar cardiovascular and nephroprotective benefits, as our reference drugs. This approach aimed to balance their protective effects on the fundamental cardiovascular and metabolic functions of patients, thus enhancing the strength of causal inference in our findings.

Why are SGLT2Is associated with increased risk of MEGI? High concentration of glucosuria may be one of the contributing factors.^21,52,58,61,64,65,66^ The external genital organs may contact with glucose in the urine after urination, and since it is often difficult to maintain adequate hygiene and dryness in the genital and perineal regions, this can further increase the risk of infection.

In subgroup analyses, we observed MEGI to be more pronounced in patients without insulin usage at baseline, patients with well-controlled diabetes (HbA_1c_ <7%), and patients with better kidney function (Table 3 and eTable 4 in Supplement 1). This phenomenon might be due to the pharmacokinetics of SGLT2Is. These agents suppress kidney glucose reabsorption in the proximal tubules, causing glucosuria. According to a study conducted by Scheen,^67^ the efficacy of SGLT2Is decreases in moderate chronic kidney disease and almost vanishes in severe chronic kidney disease. This means better kidney function results in higher concentrations of glucosuria and a better HbA_1c_ response. In other words, the more pronounced MEGI observed in the subgroup analyses may be partially explained by the better kidney function in these groups.

In subgroup analyses from CGRD, we specifically observed the MEGI risk to be more pronounced in younger patients. This may be partially explained by the inflammation status in elderly patients, or inflammaging,^65^ which is characterized by elevated inflammation related to age. Essentially, the association of SGLT2Is with increased risk of MEGI might be attenuated in older patients because of their inflammation status and multiple comorbidities, and this age-related inflammation could be an explanation for our findings. However, as we did not observe a differential treatment effect by age in the NHIRD analysis, whether younger patients are indeed more susceptible to MEGI when treated with SGLT2Is requires confirmation in larger studies. As for patients with poorer glycemic control, both heightened inflammation status and increased oxidative damage have been observed,^68,69,70^ which might also dilute the MEGI-promoting effect of SGLT2Is.

On the basis of additional analysis from the CGRD, both SGLT2Is and GLP-1RAs provided effective diabetes management and kidney function preservation over a 3-year follow-up period (eTable 5 in Supplement 1). In eTable 3 in Supplement 1 receiving care within a hospital system, the consistency of findings with those from the NHIRD—a nationwide, population-based database—enhances the reproducibility of our results. This dual-database approach balances clinical depth with population breadth and provides further confidence in the observed associations. Nonetheless, the incidence rate of MEGI was higher in the SGLT2I group than in the GLP-1RA group (3.63 cases per 1000 person-years vs 2.32 cases per 1000 person-years; HR 1.55; 95% CI, 1.28-1.89).

When stratified by follow-up duration, the risk of MEGI appeared to increase modestly over time, with HRs of 1.22 (95% CI, 0.91-1.62) at 1 year, 1.29 (95% CI, 1.02-1.62) at 2 years, and 1.34 (95% CI, 1.09-1.64) at 3 years (eTable 3 in Supplement 1). This trend suggests a potential cumulative exposure effect, in which prolonged use of SGLT2Is may be associated with a progressively elevated risk of MEGI.

The increasing trend in HRs supports the hypothesis of a time-dependent mechanism, possibly related to sustained glucosuria and its impact on local mucosal immunity or microbial balance. Taken together, these findings suggest that the increased MEGI risk is more likely attributable to the pharmacological effect of SGLT2Is themselves, rather than differences in glycemic control or kidney function preservation.

In eTable 3 in Supplement 1, a significant association emerged only after IPTW adjustment, indicating that confounding in the crude analysis, such as differences in age, comorbidities, or glycemic and kidney function, may have masked the true association. IPTW helped balance these covariates, mimicking a randomized comparison.

To further assess the potential impact of unmeasured confounding, we conducted an E-value analysis (eFigure 4 in Supplement 1). This analysis estimates the minimum strength of association that an unmeasured confounder would need to have with both the exposure (SGLT2I use) and the outcome (MEGI) to fully explain away the observed association. The calculated E-value suggests that only a relatively strong unmeasured confounder could negate our findings, thereby reinforcing the robustness and credibility of the observed association.

### Strengths and Limitations

We implemented a target trial emulation framework using a claims database and electronic medical records to enhance causal inference in our study and to provide additional empirical evidence for clinical decision-making. To comprehensively assess the risk of MEGI, we examined an integrated outcome that included balanoposthitis, scrotal infections, epididymo-orchitis, and FG. This approach complements prior literature by extending the scope of evaluation to a wider range of clinically relevant infections and incorporating laboratory-based indicators of kidney function (eGFR) and glycemic control (HbA_1C_), which were not consistently available in earlier population-based studies. Importantly, our database contains laboratory data, allowing us to account for potential confounders, such as kidney function and diabetes severity, that were not addressed in previous database studies. An elevated risk was noted in those using SGLT2Is. This was heightened among patients with better kidney function or those with better glycemic control. Maintaining proper hygiene by patients and close monitoring for symptoms of genital infections by clinicians are essential.

Our study also has some limitations. First, the NHIRD and CGRD lack information on self-paid or over-the-counter medications. However, as we implemented an active comparator study design, the proportion of self-paid or over-the-counter medications was likely nondifferential between the 2 groups. Second, MEGI outcomes were identified via *ICD-9-CM* and *ICD-10-CM* codes, which may be subject to misclassification. Third, we could not adjust for unmeasured confounders such as personal hygiene, which may be linked to occupation, socioeconomic status, and family environment. Nevertheless, the E-value analysis indicated that an unmeasured confounder would have required a minimum strength of association of 2.69 to refute our main findings.

## Conclusions

This cohort study found that the use of SGLT2Is was associated with an approximately 1.5-fold increased risk of MEGI compared with the use of GLP-1RA in male patients with T2D. This association remained consistent across observation periods ranging from 1 to 3 years. These findings highlight the importance of informing patients prescribed SGLT2I about the potential risk of MEGI and emphasizing proper hygiene practices to help prevent its occurrence.

## Notes

Supplement 1. **eTable 1.** Target Trial Emulation Framework **eTable 2.** Baseline Characteristics (CGRD) **eTable 3.** Comparative Risks of Male External Genital Infection for GLP-1 RAs vs. SGLT2Is (CGRD) **eTable 4.** Subgroup Analyses Using Data From CGRD **eTable 5.** Glycemic Control and Kidney Function Changes Post-Treatment (CGRD) **eFigure 1.** Exclusion Criteria (NHIRD) **eFigure 2.** Exclusion Criteria (CGRD) **eFigure 3.** Study Diagram **eFigure 4.** Forest Plot (NHIRD) **eFigure 5.** Forest Plot (CGRD) **eFigure 6.** Effect of Unmeasured Confounders

Supplement 2.
Data Sharing Statement
