# Supplementary material for: SGLT2 Inhibitors and External Genital Infection in Male Patients With Type 2 Diabetes
Source: JAMA Netw Open. 2025 Sep 29;8(9):e2534485. doi: 10.1001/jamanetworkopen.2025.34485 (PMC12481227; doi:10.1001/jamanetworkopen.2025.34485)
Supplement: Supplement 1. — eTable 1. Target Trial Emulation Framework eTable 2. Baseline Characteristics (CGRD) eTable 3. Comparative Risks of Male External Genital Infection for GLP-1 RAs vs. SGLT2Is (CGRD) eTable 4. Subgroup Analyses Using Data From CGRD eTable 5. Glycemic Control and Kidney Function Changes Post-Treatment (CGRD) eFigure 1. Exclusion Criteria (NHIRD) eFigure 2. Exclusion Criteria (CGRD) eFigure 3. Study Diagram eFigure 4. Forest Plot (NHIRD) eFigure 5. Forest Plot (CGRD) eFigure 6. Effect of Unmeasured Confounders [file jamanetwopen-e2534485-s001.pdf]

Supplemental Online Content

Cheng TH, Lin KC. Chuang ATM, et al. Risk of external genital infection and use of SGLT2 inhibitors in male patients with type 2 diabetes. *JAMA Network Open.* 2025;8(9):e2534485. doi:10.1001/jamanetworkopen.2025.34485

eTable 1. Target trial emulation framework

eTable 2. Baseline characteristics (CGRD)

eTable 3. Comparative risks of male external genital infection for GLP-1 RAs vs. SGLT2Is (CGRD)

eTable 4. Subgroup analyses using data from CGRD

eTable 5. Glycemic control and kidney function changes post-treatment (CGRD)

eFigure 1. Exclusion criteria (NHIRD)

eFigure 2. Exclusion criteria (CGRD)

eFigure 3. Study diagram

eFigure 4. Forest plot (NHIRD)

eFigure 5. Forest plot (CGRD)

eFigure 6. Effect of Unmeasured Confounders

This supplemental material has been provided by the authors to give readers additional information about their work.

© 2025 Cheng TH et al. *JAMA Network Open*.

eTable 1. Target trial emulation framework.

Component	Target trial	Emulated target trial
Aim	To estimate the relative effect of SGLT2Is versus GLP1-RAs on the risk of MEGI in T2DM patients	Same as target trial
Eligibility criteria (inclusion)	1. Adult male patients with T2DM<br>2. Age>=20<br>3. Patients newly received SGLT2Is or GLP1-RAs, washout period 1 year	Same as target trial
Eligibility criteria (exclusion)	1. Patients with history of organ transplantation<br>2. Patients with congenital immunodeficiency<br>3. Patients diagnosed with MEGI within one year before index date	Same as target trial
Treatment strategies	1. Receiving SGLT2Is (empagliflozin, dapagliflozin or canagliflozin)<br>2. Receiving GLP1-RAs (liraglutide or dulaglutide)	Same as target trial
Treatment assignment	Eligible patients are randomly assigned to either regimen.	Propensity score with inverse probability of treatment weighting to generate two groups with similar probability of treatment assignment.
Follow-up	Patient follow-up from the index date until the end of the trial	In the analysis using NHIRD, we follow patients from index date to the first occurrence of MEGI, death, or the last date of the database.<br>In the analysis using CGRD, we follow patients from the index date until the first occurrence of MEGI, death, last clinical visit, or the last date of the database.
Outcome	Incident MEGI event, defined as the composite outcome of balanoposthitis, scrotal infections, epididymo-orchitis, or Fournier gangrene.	Same as target trial
Causal contrast	Intention to treat effect	Same as target trial
Statistical analysis	Cox proportional hazards model	Same as target trial<br>We also conducted<br>1. subgroup analysis based on age, renal function and glycemic control<br>2. sensitivity analysis of on-treatment analysis, and different observation periods.

Abbreviations - SGLT2Is: Sodium-glucose cotransporter-2 inhibitors. GLP1-RAs: Glucagon-like peptide-1 receptor agonists, T2DM: Type 2 diabetes mellitus, MEGI: Male external genital infection

© 2025 Cheng TH et al. *JAMA Network Open*.

eTable 2. Baseline characteristics (CGRD)

Characteristic	Before IPTW trimming			After IPTW trimming		
	SGLT2Is	GLP-1 RAs	ASMD[a]	SGLT2Is	GLP-1 RAs	ASMD[a]
Number of patients	n=23849	n =2115		n=25868	n =24543	
Age, mean (SD), (year)	60.4(12.2)	59.6(13.8)	0.06	60.2(13)	58.8(46)	0.04
20-40 years (n, %)	1288(5.4%)	188(8.8%)	-0.14	1479(5.8%)	2045(8.4%)	0.10
40-60 years (n, %)	9321(39%)	824(39%)	0.00	10119(39.2%)	10985(44.8%)	0.11
60-80 years (n, %)	12038(50.4%)	955(45.2%)	0.11	12959(50%)	9832(40%)	0.20
≥80 years (n, %)	1202(5%)	148(7%)	-0.08	1312(5%)	1682(6.8%)	0.08
Laboratory data						
HbA1c, median % (IQR)	8(7-9)	9(8-10)	-0.50	8(7-9)	8(7-10)	0.01
eGFR, median (IQR), mL/min/1.73 m2	81(66-98)	71(37-96)	0.41	81(64-98)	84(66-100)	0.01
AST elevated (n, %)	1181(5%)	117(5.6%)	-0.03	1266(4.8%)	1820(7.4%)	0.10
ALT elevated (n, %)	4316(18%)	379(18%)	0.00	4654(18%)	5181(21.2%)	0.08
LDL >130 (n, %)	2715(11.4%)	258,12.2%	-0.03	2976(11.6%)	3302(13.4%)	0.06
HDL <40 (n, %)	9437(39.6%)	998(47.2%)	-0.15	10307(39.8%)	10626(43.2%)	0.07
TG > 150 (n, %)	124(0.6%)	15(0.8%)	-0.02	165(0.6%)	177(0.8%)	0.01
PT prolonged (n, %)	903(3.8%)	97(4.6%)	-0.04	1016(4%)	949(3.8%)	0
APTT prolonged (n, %)	3018(12.6%)	304(14.4%)	-0.05	3334(12.8%)	2950(12%)	0.03
Low Total testosterone (n, %)	117(0.4%)	15(0.8%)	-0.03	125(0.4%)	218(0.8%)	0.05
Elevated PSA (n, %)	276(1.2%)	33(1.6%)	-0.03	298(1.2%)	437(1.8%)	0.05
Leukocytosis (n, %)	20(0%)	2(0%)	0.00	22(0%)	25(0.2%)	0.01
Serum creatinine abnormal (n, %)	6818(28.6%)	1121(53%)	-0.51	7820(30.2%)	8410(34.2%)	0.09
Serum potassium abnormal (n, %)	929(3.8%)	160(7.6%)	-0.16	1054,4%	961(4%)	0.01
Hematocrit abnormal (n, %)	3724(15.6%)	678(32%)	-0.39	4302(16.6%)	4716(19.2%)	0.07
Risk factors of male external genital infection (MEGI)						
Congestive heart failure (n, %)	2121(8.8%)	187(8.8%)	0.00	2229(8.6%)	2026(8.2%)	0.01
Alcohol abuse (n, %)	48(0.2%)	3(0.2%)	0.01	49(0.2%)	24(0%)	0.02
Peripheral vascular disease (n, %)	100(0.4%)	25(1.2%)	-0.09	119(0.4%)	117(0.4%)	0
Hypertension (n, %)	12477(52.4%)	1225(58%)	-0.11	13658(52.8%)	12408(50.6%)	0.04

© 2025 Cheng TH et al. *JAMA Network Open*.

Obesity (n, %)	1065(4.4%)	124(5.8%)	-0.06	1194(4.6%)	1411(5.8%)	0.05
Chronic kidney disease						
CKD stage 1 (n, %)	6683(28%)	475(22.4%)	0.13	7140(27.6%)	7109(29%)	0.03
CKD stage 2 (n, %)	8244(34.6%)	411(19.4%)	0.35	8581(33.2%)	7948(32.4%)	0.02
CKD stage 3 (n, %)	2789(11.6%)	379(18%)	-0.18	3188(12.4%)	3051(12.4%)	0
CKD stage 4 (n, %)	169(0.8%)	123(5.8%)	-0.29	304(1.2%)	317(1.2%)	0.01
CKD stage 5 (n, %)	146(0.6%)	166(7.8%)	-0.37	288(1.2%)	286(1.2%)	0
Coagulopathy (n, %)	22(0%)	3(0.2%)	-0.01	24(0%)	10(0%)	0.02
Liver failure (n, %)	155(0.6%)	15(0.8%)	-0.01	168(0.6%)	115(0.4%)	0.02
Major adverse cardiac event						
Myocardial infarction (n, %)	1451(6%)	103(4.8%)	0.05	1493(5.8%)	1275(5.2%)	0.03
Stroke (n, %)	2281(9.6%)	253(12%)	-0.08	2530(9.8%)	2908(11.8%)	0.07
Unstable angina (n, %)	272(1.2%)	13(0.6%)	0.06	261(1%)	140(0.6%)	0.05
Diabetes comedication						
Metformin (n, %)	17784(74.6%)	1196(56.6%)	0.39	18911(73.2%)	17267(70.4%)	0.06
Sulfonylurea (n, %)	10985(46%)	1136(53.8%)	-0.15	12145(47%)	11330(46.2%)	0.02
Dipeptidyl peptidase-4 inhibitor (n, %)	13146(55.2%)	1443(68.2%)	-0.27	14566(56.4%)	12861(52.4%)	0.08
Thiazolidinedione (n, %)	3224(13.6%)	375(17.8%)	-0.12	3624(14%)	3668(15%)	0.03
Alpha-reductase inhibitor (n, %)	2784(11.6%)	416(19.6%)	-0.22	3213(12.4%)	3270(13.4%)	0.03
Glinide (n, %)	603(2.6%)	171(8%)	-0.25	772(3%)	803(3.2%)	0.02
Insulin (n, %)	3949(16.6%)	1166(55.2%)	-0.88	5130(19.8%)	5345(21.8%)	0.05

Abbreviations - CGRD: Chang Gung Research Database, IPTW: inverse probability of treatment weighting, SD: standard deviation, HbA1c: hemoglobin A1c, IQR: interquartile range, eGFR: estimated glomerular filtration rate, AST: aspartate Transaminase, ALT: alanine aminotransferase, LDL: low-density lipoprotein, HDL: high-density lipoprotein, TG: triglycerides, PT: prothrombin time, APTT: Activated Partial Thromboplastin Time, PSA: prostate-specific antigen, BPH: benign prostate hyperplasia, GLP-1 RAs: glucagon-like peptide-1 receptor agonists, SGLT2Is: sodium-glucose cotransporter-2 inhibitors, ASMD: absolute standardized mean difference.

[a] Values above 0.1 suggest a meaningful difference between the two treatment groups.

© 2025 Cheng TH et al. *JAMA Network Open*.

eTable 3. Comparative risks of male external genital infection for GLP-1 RAs vs. SGLT2Is (CGRD)

Variable	Patients	Incidence rate (95% CI) per 1000 person-years	HR (95% CI)
Crude analysis			
GLP-1 RAs	2115	3.17 (1.91-4.95)	1.00[Reference]
SGLT2Is	23849	3.57 (3.12-4.06)	1.12 (0.7-1.79)
Main analysis (IPTW)			
GLP-1 RAs	2093	2.32 (1.98-2.7)	1.00[Reference]
SGLT2Is	23746	3.63 (3.2-4.11)	1.55 (1.28-1.89)
Sensitivity analysis			
1:1 propensity score matching			
GLP-1 RAs	1968	3.03 (1.76-4.85)	1.00[Reference]
SGLT2Is	1968	3.99 (2.47-6.1)	2.2 (0.76-6.33)
On-treatment analysis			
GLP-1 RAs	2093	1.56 (1.2-1.99)	1.00[Reference]
SGLT2Is	23746	3.18 (2.7-3.71)	2.07 (1.55-2.76)
Different follow-up periods			
1 year			
GLP-1 RAs	2093	3.66 (2.92-4.53)	1.00[Reference]
SGLT2Is	23746	4.45 (3.64-5.37)	1.22 (0.91-1.62)
2 years			
GLP-1 RAs	2093	3.03 (2.52-3.6)	1.00[Reference]
SGLT2Is	23746	3.9 (3.33-4.53)	1.29 (1.02-1.62)
3 years			
GLP-1 RAs	2093	2.87 (2.44-3.35)	1.00[Reference]
SGLT2Is	23746	3.84 (3.35-4.39)	1.34 (1.09-1.64)

Abbreviations - CGRD: Chang Gung Research Database, GLP-1 RAs: glucagon-like peptide-1 receptor agonists, SGLT2Is: sodium-glucose cotransporter-2 inhibitors, CGRD: Chang Gung Research Database, IPTW: inverse probability of treatment weighting, CI: confidence interval, HR: hazard ratio.

© 2025 Cheng TH et al. *JAMA Network Open*.

eTable 4. Subgroup analyses using data from CGRD.

Variable	Patients	Incidence rate (95% CI) per 1000 person-years	HR (95% CI)
Age			
<60			
GLP-1 RAs	996	2.25 (1.81-2.77)	1[Reference]
SGLT2Is	10587	4.71 (3.99-5.51)	2.04 (1.58-2.65)
≥60			
GLP-1 RAs	1097	2.40 (1.88-3.02)	1[Reference]
SGLT2Is	13159	2.68 (2.18-3.27)	1.13 (0.84-1.53)
eGFR level (mL/min/1.73 m2)			
<60			
GLP-1 RAs	646	2.74 (1.85-3.92)	1[Reference]
SGLT2Is	3103	2.73 (1.79-3.99)	1.03 (0.61-1.75)
≥60			
GLP-1 RAs	886	2.31 (1.9-2.79)	1[Reference]
SGLT2Is	14832	3.97 (3.41-4.59)	1.69 (1.34-2.15)
HbA1c level (%)			
<7%			
GLP-1 RAs	177	1.07 (0.58-1.82)	1[Reference]
SGLT2Is	4334	3.48 (2.44-4.81)	3.22 (1.71-6.03)
≥7%			
GLP-1 RAs	1745	2.11 (1.74-2.53)	1[Reference]
SGLT2Is	17605	3.64 (3.15-4.17)	1.72 (1.37-2.16)

Abbreviations - CGRD: Chang Gung Research Database, SGLT2Is: sodium-glucose cotransporter-2 inhibitors, GLP-1 RAs: glucagon-like peptide-1 receptor agonists, eGFR: estimated glomerular filtration rate, HbA1c: hemoglobin A1c, CI: confidence interval, HR: hazard ratio

© 2025 Cheng TH et al. *JAMA Network Open*.

eTable 5. Glycemic control and kidney function changes post-treatment (CGRD)

Variable	SGLT 2Is	GLP-1 RAs	ASMD[a]
Baseline HbA1c, median (IQR), %	8(7-9)	8(7-10)	0.01
HbA1c, median (IQR), %			
1st year	7.2(6.6-8.1)	7.4(6.4-8.4)	0.03
2nd year	7.2(6.6-8)	7.2(6.5-8.3)	0.03
3rd year	7.2(6.6-8)	7.2(6.6-8.4)	0.03
Baseline eGFR, median (IQR), mL/min/1.73 m2	81(64-98)	84(66-100)	0.01
eGFR, median (IQR), mL/min/1.73 m2			
1st year	79(62-97)	83(62-98)	0.00
2nd year	79(62-95)	80(63-95)	0.01
3rd year	80(63-96)	80(59-95)	0.03

Abbreviations - CGRD: Chang Gung Research Database, IPTW: inverse probability of treatment weighting, HbA1c: hemoglobin A1c, IQR: interquartile range, eGFR: estimated glomerular filtration rate, SGLT2Is: sodium-glucose cotransporter-2 inhibitors, GLP-1 RAs: glucagon-like peptide-1 receptor agonists, ASMD: absolute standardized mean difference.

[a] Values above 0.1 suggest a meaningful difference between the two treatment groups.

.

© 2025 Cheng TH et al. *JAMA Network Open*.

eFigure 1. Exclusion criteria (NHIRD).

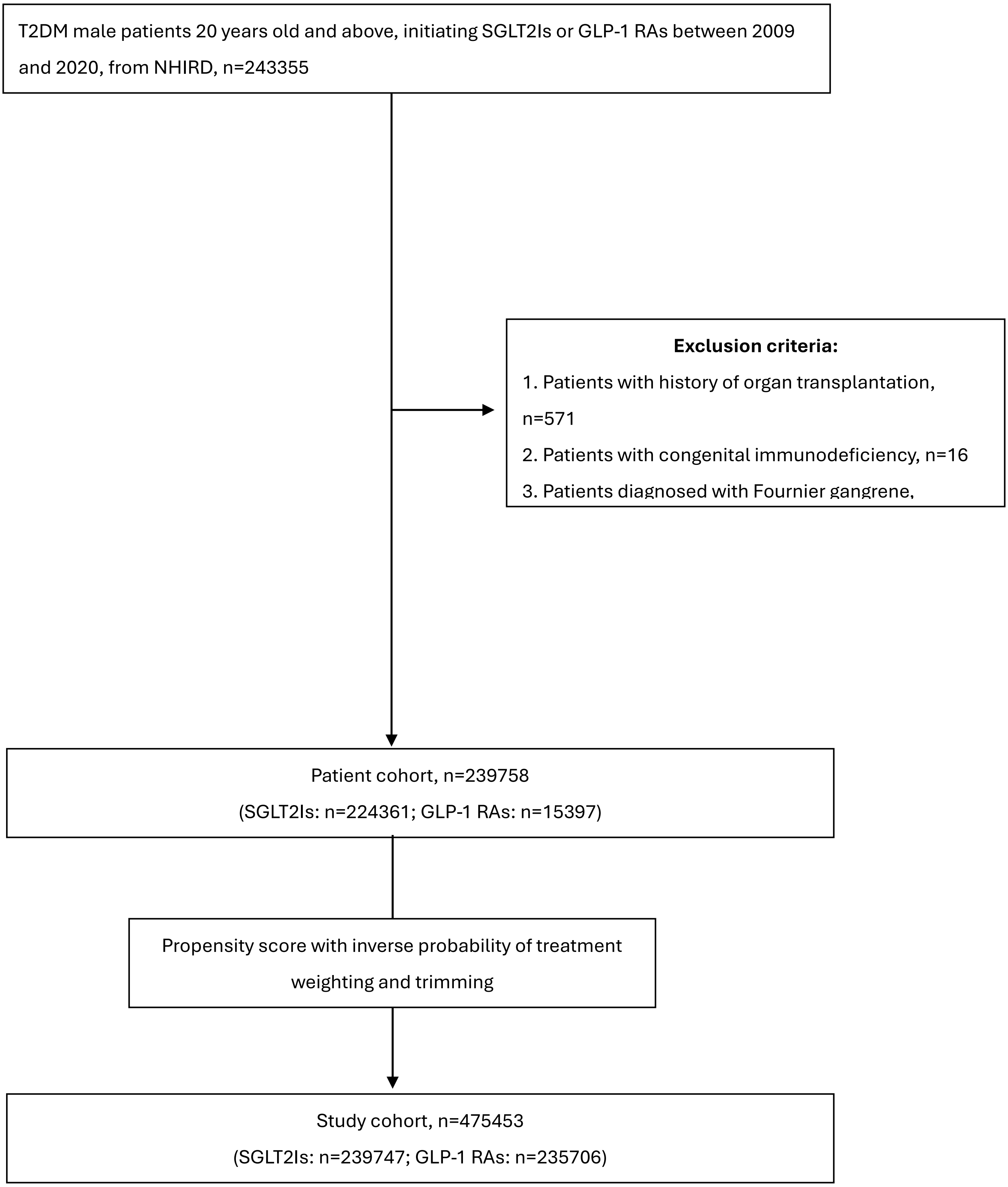

Abbreviations - NHIRD: National Health Insurance Research Database, T2DM: type 2 diabetes mellitus, SGLT2Is: sodium-glucose cotransporter-2 inhibitors, GLP1-RAs: glucagon-like peptide-1 receptor agonists, IPTW: inverse probability of treatment weighting.

© 2025 Cheng TH et al. *JAMA Network Open*.

eFigure 2. Exclusion criteria (CGRD).

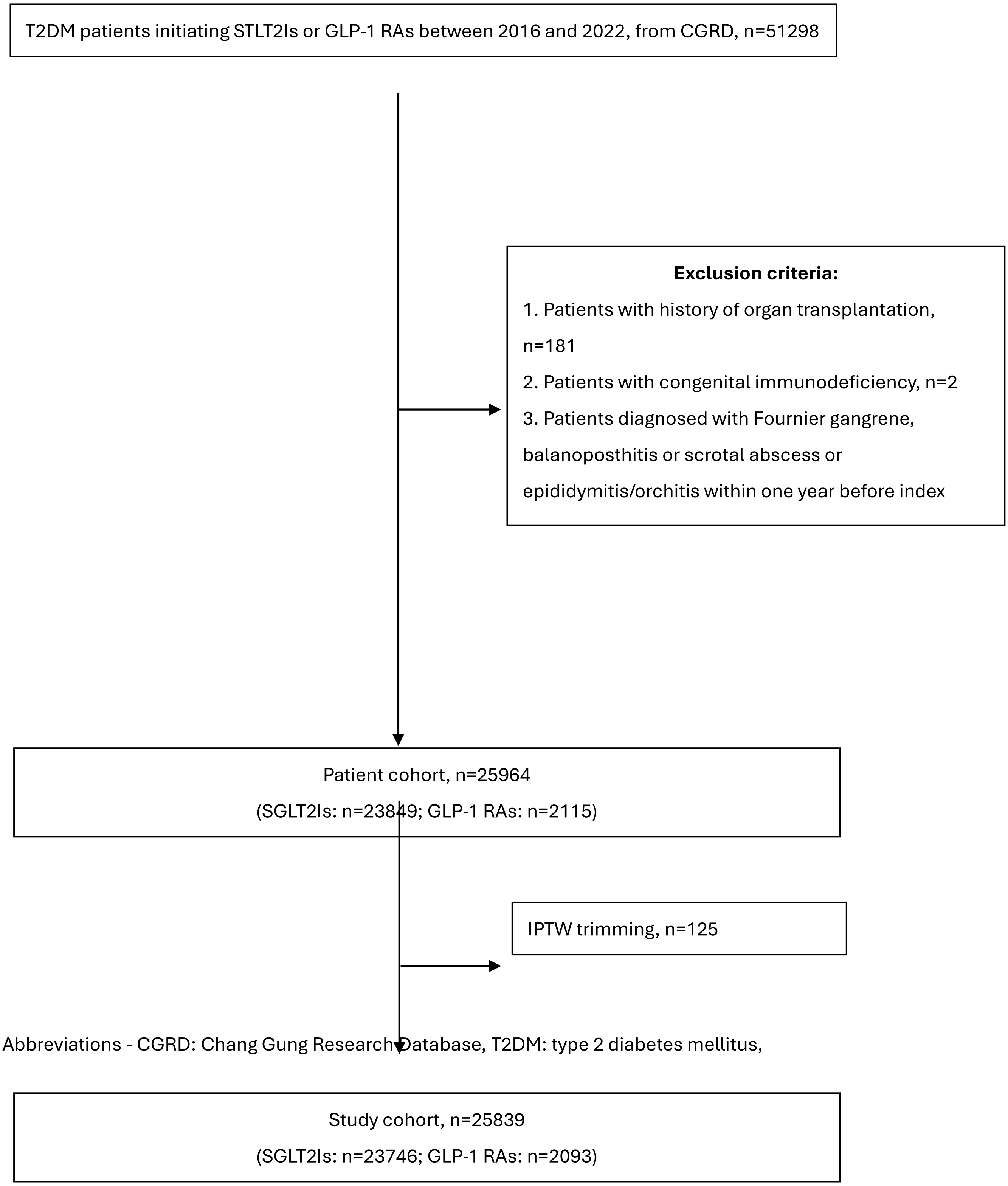

Abbreviations - CGRD: Chang Gung Research Database, T2DM: type 2 diabetes mellitus, SGLT2Is: sodium-glucose cotransporter-2 inhibitors, GLP1-RAs: glucagon-like peptide-1 receptor agonists, CGRD: Chang Gung Research Database, IPTW: inverse probability of treatment weighting.

© 2025 Cheng TH et al. *JAMA Network Open*.

eFigure 3. Study diagram

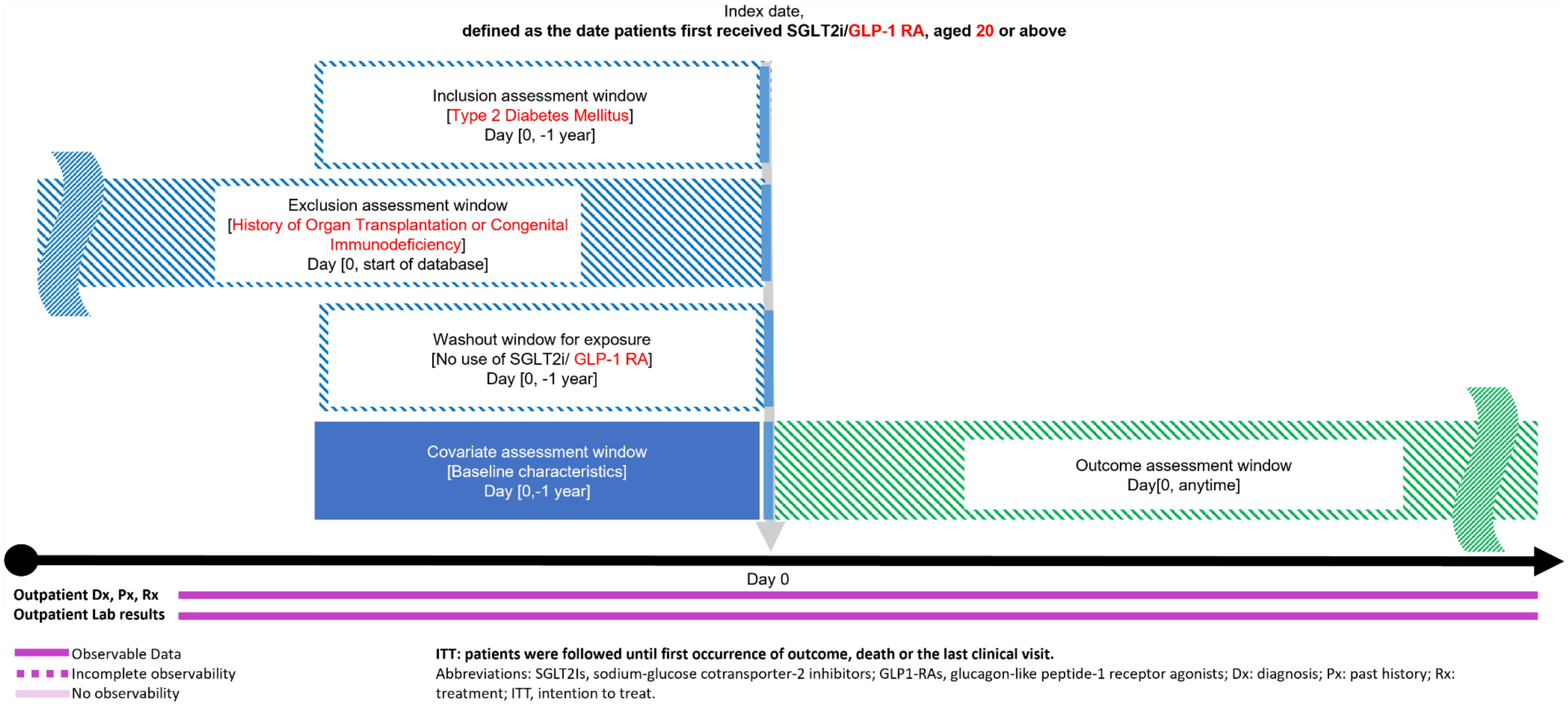

ITT: patients were followed until first occurrence of outcome, death or the last clinical visit.

Abbreviations: SGLT2Is, sodium-glucose cotransporter-2 inhibitors; GLP1-RAs, glucagon-like peptide-1 receptor agonists; Dx: diagnosis; Px: past history; Rx: treatment; ITT, intention to treat.

© 2025 Cheng TH et al. *JAMA Network Open*.

eFigure 4. Forest plot (NHIRD)

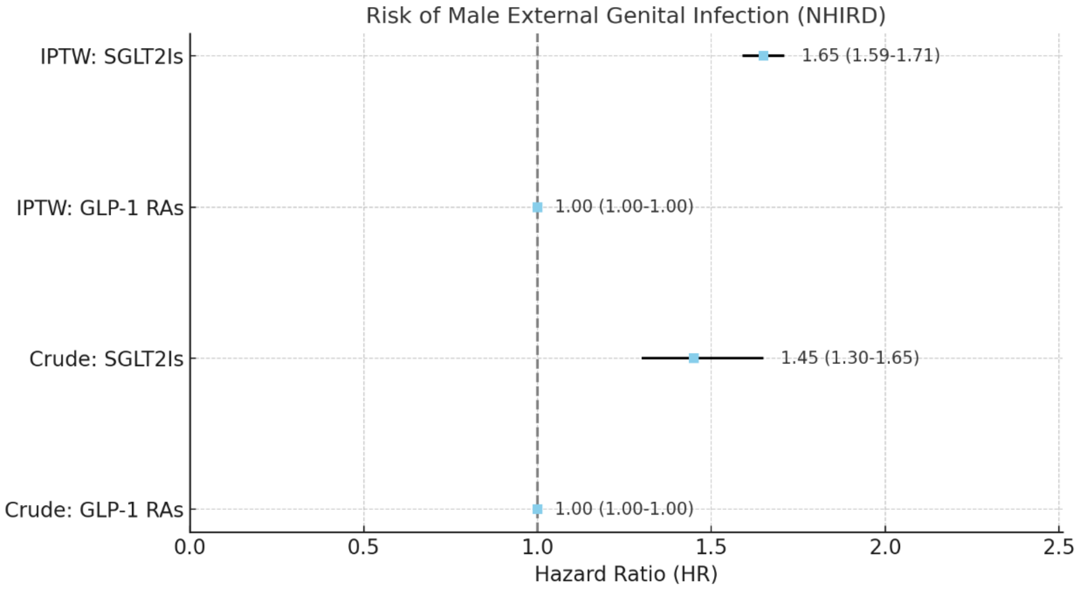

© 2025 Cheng TH et al. *JAMA Network Open*.

eFigure 5 Forest plot (CGRD)

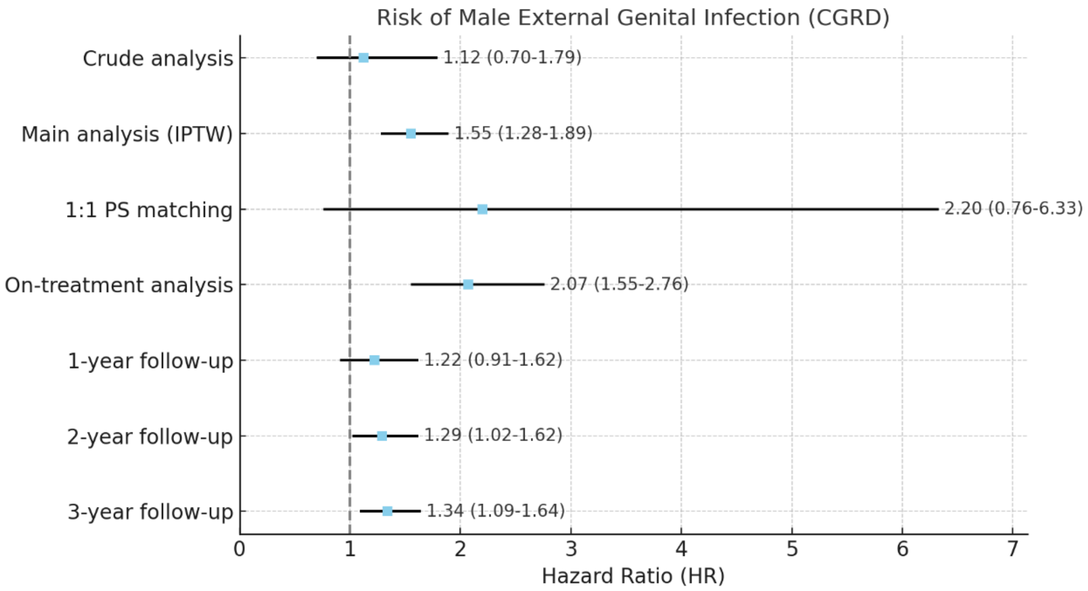

© 2025 Cheng TH et al. *JAMA Network Open*.

eFigure 6. Effect of Unmeasured Confounders.

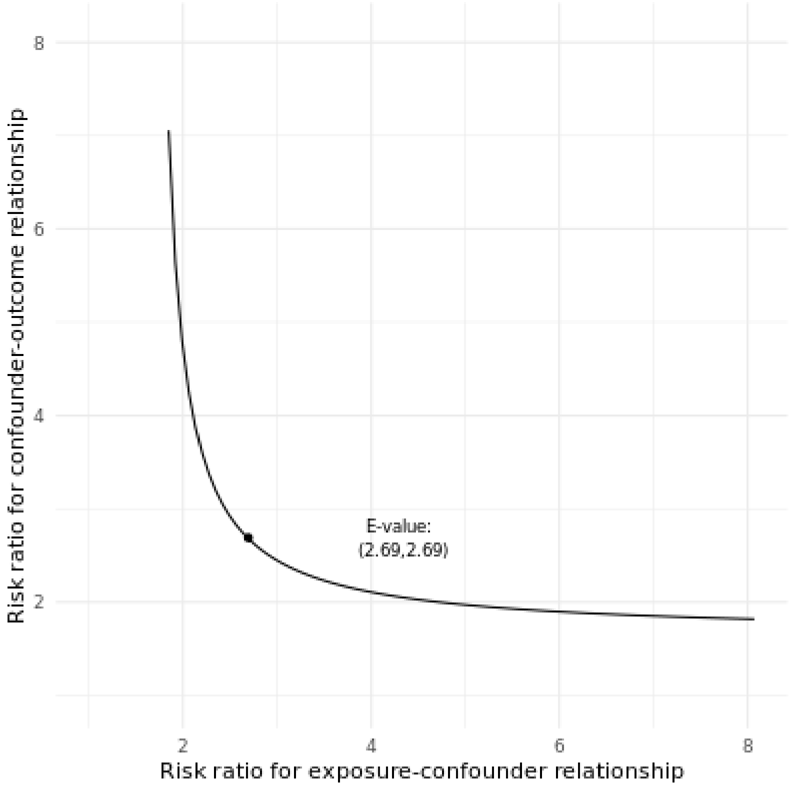

Each point along the curve represents a joint relationship between the two sensitivity parameters that could potentially account for the estimated effect. If one of the two parameters is less than the E-value, the other must be greater, as indicated by the plotted curve.

© 2025 Cheng TH et al. *JAMA Network Open*.
