# Supplementary material for: SGLT2 Inhibitors and External Genital Infection in Male Patients With Type 2 Diabetes
Source: JAMA Netw Open. 2025 Sep 29;8(9):e2534485. doi: 10.1001/jamanetworkopen.2025.34485 (PMC12481227; doi:10.1001/jamanetworkopen.2025.34485)
Supplement: Supplement 2. — Data Sharing Statement [file jamanetwopen-e2534485-s002.pdf]

Data Sharing Statement

Cheng. Risk of External Genital Infection and Use of SGLT2 Inhibitors in Male Patients With Type 2 Diabetes. *JAMA Netw Open*. Published September 29, 2025. doi:10.1001/jamanetworkopen.2025.34485

Data
Data available: No

Additional Information
Explanation for why data not available: The data used in this study are from Taiwan's National Health Insurance Research Database, which is publicly unavailable for data sharing due to legal and ethical restrictions. Interested researchers may apply for access through the appropriate application process.
